# Hybrid Model-Based Simulation Analysis on the Effects of Social Distancing Policy of the COVID-19 Epidemic

**DOI:** 10.3390/ijerph182111264

**Published:** 2021-10-27

**Authors:** Bong Gu Kang, Hee-Mun Park, Mi Jang, Kyung-Min Seo

**Affiliations:** 1 Research Institute of Industrial Technology Convergence, Korea Institute of Industrial Technology (KITECH), Ansan 15588, Korea; bgkang@kitech.re.kr; 2 Department of Computer Engineering, Korea University of Technology and Education (KOREATECH), Cheonan 31253, Korea; phm1321@koreatech.ac.kr (H.-M.P.); dntdmami@koreatech.ac.kr (M.J.); 3 Department of Future Technology, Korea University of Technology and Education (KOREATECH), Cheonan 31253, Korea

**Keywords:** simulation, SIRD model, discrete-event model, data-based learning, COVID-19 epidemic

## Abstract

This study utilizes modeling and simulation to analyze coronavirus (COVID-19) infection trends depending on government policies. Two modeling requirements are considered for infection simulation: (1) the implementation of social distancing policies and (2) the representation of population movements. To this end, we propose an extended infection model to combine analytical models with discrete event-based simulation models in a hybrid form. Simulation parameters for social distancing policies are identified and embedded in the analytical models. Administrative districts are modeled as a fundamental simulation agent, which facilitates representing the population movements between the cities. The proposed infection model utilizes real-world data regarding suspected, infected, recovered, and deceased people in South Korea. As an application, we simulate the COVID-19 epidemic in South Korea. We use real-world data for 160 days, containing meaningful days that begin the distancing policy and adjust the distancing policy to the next stage. We expect that the proposed work plays a principal role in analyzing how social distancing effectively affects virus prevention and provides a simulation environment for the biochemical field.

## 1. Introduction

During the initial outbreak of coronavirus disease (COVID-19), the major propagation factors were population movement and cluster infections in groups [1,2,3]. In South Korea, for example, several situations exploded due to carriers in groups such as churches and hospitals [4]. In Singapore, a serological survey of citizens who traveled abroad confirmed an early spreader [5]. As one of the solutions for COVID-19 prevention, governments worldwide implemented social distancing to prevent the spread of COVID-19. The United States asserted the spreading speed in states conducting social distancing was six times slower than those that did not [6].

To analyze how social distancing is effective in COVID-19 prevention, we utilize modeling and simulation (M&S) methods in this study. Although simulations containing population movement are intrinsically complicated, well-categorized models are understandable, analyzable, and certifiable [7,8,9,10]. Therefore, they give helpful insights to analyze and predict COVID-19 situations [11,12,13]. Based on real-world data regarding confirmed cases in South Korea, the modeling requirements for the COVID-19 simulation are twofold: (1) the movement of the population and (2) the implementation of social distancing policies. 

Various M&S methods for the COVID-19 epidemic have been conducted during the past two years. They overcome the limitations of analytical and simulation models. Some researchers have proposed analytical methods with several infection types [14,15,16], and others have developed simulation models over the partial region [17,18]. The collective contribution of these studies is to suggest meaningful modeling methods and simulation results for the virus infection. Despite the contribution, they still warrant some improvements. For example, some analytical models are limited in reflecting events, such as policies and population movements; others have focused on the microscopic or macroscopic region, which results in difficulty in conducting the simulation-based analysis at the national level. 

This study presents a practical M&S approach to analyze the trends of the COVID-19 epidemic. The proposed model consists of two steps to overcome the limitations of analytical and simulation models: (1) hybrid infection model design and (2) model identification. The proposed infection model utilized real-world data regarding suspected, infected, recovered, and deceased people in South Korea. Based on the data, we design an analytical model known as the SIRD (Susceptible, Infected, Recovered, and Dead) model [16]. Then, we complete an overall simulation model to combine a discrete event model with the SIRD model in a hybrid form. Model coefficients within the overall model contain domestic policies, such as social distancing and regional population movements. We optimized the developed model by updating the coefficients by stages to make the model reflect real-world data.

From a simulation modeling view, we considered an administrative district in South Korea as a fundamental agent. In other words, simulation agents were set in units of administrative districts to express virus transmission within and between the regions. Two main reasons for this simulation modeling are as follows. First, if we considered one person as an agent, as the number of people to be analyzed expanded, simulation agents would also increase, and it may take longer to acquire simulation results. Next, district-level agent modeling helped reflect population movement between the districts. 

As a case study, we simulated the COVID-19 epidemic in South Korea to analyze the real-world infection trend and the changes in government policy. A social distancing policy in South Korea is classified into four levels according to the number of confirmed cases per 100,000 people. For infection prevention, the policy compels two primary factors: movement and gathering. For example, in the third level of social distancing, up to four people are permitted for a private gathering. Wearing a mask is not included in the policy; however, South Korea strongly encourages people to wear a mask. In this simulation, we assume that people correctly put on a mask. We used real-world data for 160 days for simulation experiments, containing two meaningful days: (1) a day to begin the distancing policy and (2) a day to adjust the distancing policy to the next stage. Seventeen administrative districts were modeled, and real-world data were used based on the population movement by region announced by the National Statistical Office of South Korea. The simulation results provide insights into pulling and delaying the enforcement of the distance policy.

This study is organized as follows. Section 2 explains related works for virus infection simulation. Section 3 proposes the overall architecture with a hybrid modeling approach, and Section 4 explains and discusses simulation experiments. Finally, Section 5 presents our conclusions.

## 2. Related Works

For the last couple of years, various studies have been conducted to provide simulation analyses for virus infections, such as the COVID-19 pandemic. In this section, we categorize them into modeling aspects, which are summarized in Table 1.

During virus outbreaks, the population can be classified mainly into five types: (1) susceptible people before infection (S), (2) people exposed to the virus (E), (3) infected people (I), (4) people who have recovered (R), and (5) people who have died (D). These types are used to design analytical models for viral infection analyses

Among the analytical modeling approaches, two primary models have been studied: the SIR model, with S, I, and R factors [14,19,20,21], and the SEIR model, which adds E factor from the SIR model [15,22,23,24]. For example, Igor utilized the SIR model to analyze the COVID-19 infection and recovery rates [14]. Because this is an early study that occurred in South Korea, the analysis period is relatively short. Mulder [15] used the SEIR model to analyze the infection exposure, infection, and recovery rates in 36 countries. Mulder’s study also cannot analyze the mortality rate. Moreover, the number of exposed people is an inappropriate model for our study based on real-world data because precise real-world data cannot be obtained. The mortality rate also could not be considered.

To improve the SIR model, the SIRD models have been studied, which contain the D factor (i.e., the number of dead people) [16,25,26,27]. For example, Calafiore et al. [16] predicted the number of S, I, R, and D factors. There is also a modified SIRD model that is more advanced than SIRD. In the case of the previous Ebola virus epidemic, there have been many cases of reinfection after recovery. Therefore, the analysis with the modified SIRD model was suitable for the Ebola virus study [28]. Similar to Calafiore et al. [16], Osemwinyen et al. [28], Basti et al. [29], Shringi et al. [30], and Devosmita et al. [31] were unable to reflect on policy and population movement. Furthermore, in the case of COVID-19, reinfection cases are sporadic in Korea; thus, a modified SIRD model was considered inappropriate.

**Table 1:** Related works for modeling and simulation studies for virus infection.

Related Work	Modeling Approach	Modeling Method	Description
[14]	Analytical model	Equation-based model (SIR)	- COVID-19 application - Derive the infection rate and recovery rate in Korea - Short analysis period (13 days) - Analysis of mortality is impossible.
[15]	Analytical model	Equation-based mode (SEIR)	- COVID-19 application - Analysis of 36 countries in which COVID-19 has been widely transmitted, including Korea - Derive the probability of exposure to infection, infection rate, and recovery rate - No real-world data can be obtained for the number of people exposed to infection.
[16]	Analytical model	Equation-based model (SIRD)	- COVID-19 application - Derive the infection rate, recovery rate, and mortality rate in Italy - Trend prediction is possible, but special events, such as policy and outbreak of group infection, cannot be reflected.
[28]	Analytical model	Equation-based model (Modified SIR)	- Ebola application - Mathematical analysis of the spread of Ebola in developing countries - Using a modified SIRD model by including reinfection in a typical SIRD model - Model unsuitable for COVID-19 with few cases of reinfection
[17]	Simulation model	Agent-based model	- COVID-19 application - Predict the effectiveness of social distancing policies in the United States
[18]	Simulation model	Agent-based model	- COVID-19 application - Predict infection among people living together in closed spaces (e.g., offices and buildings)

Equation-based models are limited in reflecting events, such as policies and population movements. Simulation models have been used to overcome the limitations of equation-based models. For example, Alagoz et al. [17] predicted the cumulative number of confirmed cases according to the date when the social distancing policy was implemented in three regions of the United States. They predicted how the cumulative number of infected people would change when the onset of social distancing was accelerated or delayed by a week. They reflected the policy in the simulation, but not the population movement between regions.

D’Orazio et al. [18] also predicted the trend of virus transmission between humans in a dense space. Although it is possible to analyze the transmission of viruses between humans, there is a limit to targeting a confined space without the population movement. However, when a person is set as an agent, as in D’Orazio et al., it is difficult to expand the analysis scope to the country. When analyzing a confined space without external inflow, it is impossible to express the propagation characteristics of COVID-19 spreading between regions.

To overcome the limitations of analytical and simulation models, we propose a hybrid modeling approach (i.e., simulation models containing the analytical models). The proposed model reflects the social distancing policy, as well as the population movements between regions. We add simulation parameters in the analytical SIRD model to reflect the social distancing policy. We divide administrative districts into a simulation agent, which facilitates representing the population movements between the cities.

## 3. Proposed Work

### 3.1. Overall Process Description

**Figure 1:**
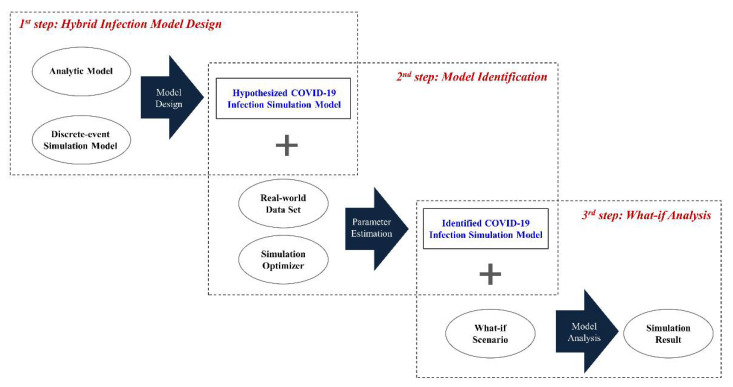
Overall process of proposed simulation model.

For the simulation-based what-if analysis (the third step in Figure 1)—the ultimate goal of this study—it is necessary to construct the simulation model by reflecting the real-world environment, which consists of two steps: (1) hybrid infection model design (first step) and (2) model identification (the second step). 

The existing simulation models for describing the infection spread process can be categorized into two types: (1) analytical model and (2) discrete-event simulation model [32]. The former focuses on the transmission of infection within a large-scale community using a differential equation-based model (EBM), which can analyze the tendency of infection at a macro level but has the disadvantage of not considering the relationship with other communities. On the other hand, the latter can describe the interaction with other communities using an agent-based model (ABM), but it is limited to a large-scale community.

In the model design step, we constructed a hybrid model to simulate infection, including interactions on a large scale, by combining two model types: the SIRD model (i.e., the analytical model) and the infection discrete-event simulation model expressed in the DEVS (discrete event systems specification) formalism and implemented in the DEVSim++ environment [33,34]. The former describes the infection behavior in the inner community, and the latter depicts the infection interaction among communities.

Although we constructed the simulation model at the previous stage, it is impossible to identify all the parameter values in the model. For example, in Table 2, we can know the duration time of the relaxed social distancing policy ($t_{rsd}$) in the policy parameter set based on the real-world data, but it is impossible to know the exact rate of infection (β) in the infection parameter set in the SIRD model. In the model identification step, to solve this problem, we identified the constructed infection simulation model using a data set acquired from the real-world and simulation optimizer [35], which calibrates the parameters in the simulation model using the optimization algorithms [36,37,38].

In the final step, we can conduct the what-if analysis using the identified infection simulation model. For example, we can analyze trends of the S(t), I(t), R(t), and D(t) against the policy parameter set of Table 3. This study deals with the first step in the following subsection and the other steps in Section 4.

### 3.2. Hybrid Infection Simulation Model Design

**Figure 2:**
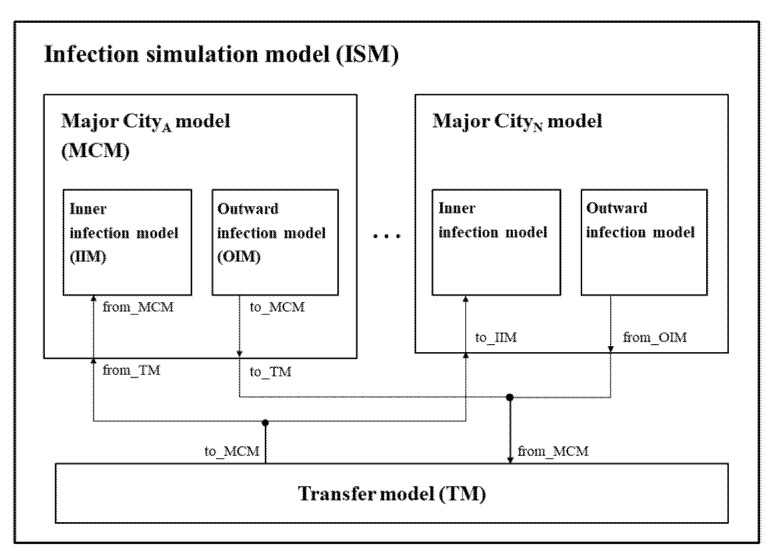
DEVS-coupled model diagram: Infection simulation model (*ISM*).

Figure 2 shows the overall architecture of the proposed hybrid infection simulation model (*ISM*). The *ISM* is a discrete-event simulation model, which includes the analytical model as an algorithm. The entire model consists of two sub-models: (1) major city model (*MCM)* and (2) transfer model (*TM*). The *MCM* and *TM* describe the metropolis city and their interaction exchanges, respectively. In the real world, because the infection spread between agents has a discrete-event property and the cities have a modular property, we expressed the entire model using the DEVS formalism. The DEVS expression of *ISM, MCM,* and *TM* are represented by Equations (1)–(25). The TM has a role in routing the infection spread from one MCM to the other MCM, and Equation (7) means their sending and receiving connection. In addition, to prioritize this role, we gave priority to the model as Equation (8).

(1) ISM=<X, Y,M, EIC, EOC, IC,Sel> 

(2) X=∅;

(3) Y=∅;

(4) $$M=\left\{TM\right\}\cup \cup_{i=1}^{n}\left\{MCM_{i}\right\};$$

(5) EIC=∅;

(6) EOC=∅;

(7) $$IC=\left\{\left(MCM_{i}.to\_TM,\;TM.from\_MCM\right),\;\left(TM.to\_MCM,MCM_{i}.from\_TM\right)\right\};$$

(8) $$Sel\left(\left\{TM,\cup_{i=1}^{n}\left\{MCM_{i}\right\}\right\}\right)=TM.$$

The type of infection spread can be categorized into two cases: within one community and from other communities [39]. Furthermore, because the time interval in which the infection is internally updated in the community can differ from events externally occurring from other communities, we separate the *MCM* model into two atomic models: the inner infection model (*IIM*) and the outward infection model (*OIM*) [40]. The *IIM* describes the inner infection using the extended SIRD model, and the *OIM* describes the moving behaviors between models based on real-world mobility data.

(9) MCM=〈X, Y, M, EIC, EOC, IC, Sel〉

(10) X={from_TM} 

(11) Y={to_TM} 

(12) M={IIM, OIM} 

(13) $$EIC=\left\{\left(MCM.from_{TM},\;IIM.from\_MCM\right)\right\}\;$$

(14) $$EOC=\left\{\left(OIM.to_{MCM},MCM.to\_TM\right)\right\}\;$$

(15) IC=∅ 

(16) Sel({IIM,OIM})=OIM 

(17) $$TM=<X,\;Y,S,\;\delta_{ext},\;\delta_{int},\;\lambda ,ta>$$

(18) X={from_MCM};

(19) Y={to_MCM};

(20) S={WAIT, SEND};

(21) $$\delta_{ext}:\left(WAIT\right)\times \left(from\_MCM\right)\to \left(SEND\right);$$

(22) $$\delta_{int}:\left(SEND\right)\to \left(WAIT\right);$$

(23) λ:(SEND)→(to_MCM).;

(24) ta:(SEND)→0,

(25)  (WAIT)→∞.

**Figure 3:**
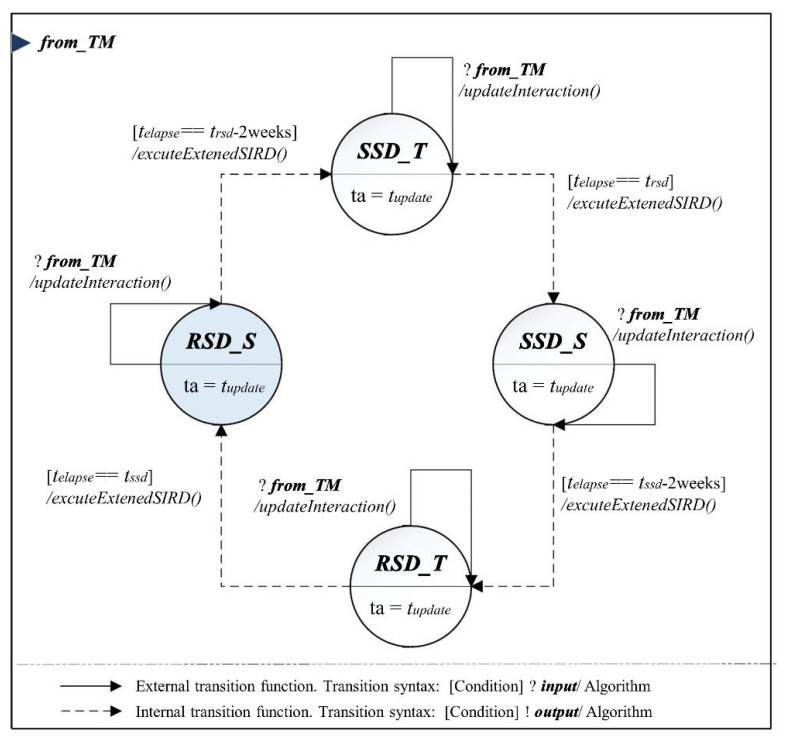
DEVS-atomic model diagram: Inner infection model (*IIM*).

Many countries have recently implemented policies to prevent the rapid spread of COVID-19 (e.g., social distancing). Despite the apparent effects [41,42], countries have intermittently used social distancing due to the social and economic impacts [43,44,45,46]. To reflect this dynamic political change and its delayed time until the effect occurs, we designed the *IIM* by separating four phases: relaxed social distancing transient and steady-state (*RSD_T*, *RSD_S*) and severe social distancing transient and steady-state (*SSD_T* and *SSD_S*), as depicted in Figure 3. The DEVS expression of the designed model is described in Equations (26)–(33). The model internally refreshes the S(t), I(t), R(t), and D(t) through the *executeExtendSIRD()* at the regular interval (i.e., $t_{update}$) in each phase, updating infection from the other model by dealing with the *from_TM* event. Then, the model transits to another phase after $t_{rsd}$ or $t_{ssd}$ about two weeks.

(26) $$IIM=<X,\;Y,S,\;\delta_{ext},\;\delta_{int},\;\lambda ,ta>\;$$

(27) X={from_TM};

(28) Y=∅;

(29) S={RSD_T,  RSD_S,  SSD_T,  SSD_S} 

(30) $$\;\delta_{ext}:\{\begin{array}{c} \left(RSD\_T\right)\times \left(from\_TM\right)\to \left(IF\right)\;and\;execute\;updateInteraction()\\ \left(RSD\_S\right)\times \left(from\_TM\right)\to \left(IF\right)\;and\;execute\;updateInteraction()\\ \left(SSD\_T\right)\times \left(from\_TM\right)\to \left(IF\right)\;and\;execute\;updateInteraction()\\ \left(SSD\_S\right)\times \left(from\_TM\right)\to \left(IF\right)\;and\;execute\;updateInteraction() \end{array}.$$

(31) $$\;\delta_{int}:\{\begin{array}{c} \left(RSD_{T}\right)\to \left(SSD_{T}\right)\;\;and\;execute\;executeExtendSIRD(),\;\\ if\;t_{e}==(t_{rsd}-2weeks)\\ \left(SSD_{T}\right)\to \left(SSD_{S}\right)\;and\;execute\;executeExtendSIRD(),\\ if\;t_{e}==t_{rsd}\\ \left(SSD_{S}\right)\to \left(RSD_{T}\right)\;and\;execute\;executeExtendSIRD(),\;\\ if\;t_{e}==(t_{ssd}-2weeks)\\ \left(RSD_{T}\right)\to \left(RSD_{S}\right)\;and\;execute\;executeExtendSIRD(),\\ if\;t_{e}==t_{ssd} \end{array}.$$

(32) λ: ∅;

(33) $$ta:\;\{\begin{array}{c} \left(RSD\_T\right)\to t_{update}\\ \left(RSD\_S\right)\to t_{update}\\ \left(SSD\_T\right)\to t_{update}\\ \left(SSD\_S\right)\to t_{update} \end{array}\;$$

To be specific, the moment a social distancing policy starts, the number of infected individuals does not immediately decrease but begins to decrease after a certain period, approximately two weeks [47]. In this study, we defined these transient and steady-state regions, respectively. To reflect this dynamic factor over time, this study extends the existing SIRD model corresponding to Equations (35)–(37). Equations (38) and (39) reflect the proposed infection rates (i.e., $\beta_{i}$ and $\beta_{d}$) over the remaining time ($t_{e}$) in the *RSD* or *SSD* phase using the attenuation coefficients (i.e., λ and κ), which are mapped in Equations (34) and (35).

(34) $$\frac{dS\left(t\right)}{dt}=\{\begin{array}{ll} -\beta_{last}\frac{SI}{N},\;\; & if\;state==RSD\_S\\ -\beta_{l}\frac{SI}{N},\;\; & if\;state==SSD\_T\\ -\beta_{d}\frac{SI}{N},\;\; & otherwise\; \end{array}\;$$

(35) $$\frac{dI\left(t\right)}{dt}=\beta \frac{SI}{N}-\gamma I-\mu \;$$

(36) $$\frac{dR\left(t\right)}{dt}=\gamma I\;\;$$

(37) $$\frac{dD\left(t\right)}{dt}=\mu I\;$$

(38) $$\beta_{i}\left(t\right)=\beta_{last}\times exp^{\lambda \left(t_{e}\right)}\;$$

(39) $$\beta_{d}\left(t\right)=\beta_{last}\times exp^{-\kappa \left(t_{e}\right)}\;$$

**Figure 4:**
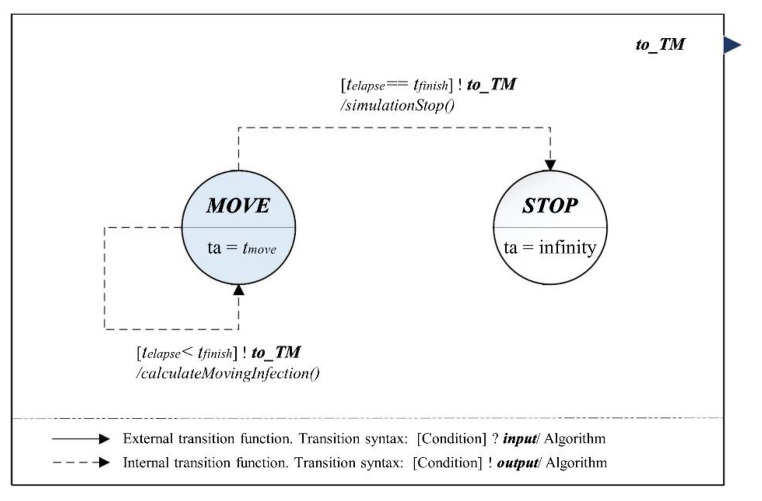
DEVS-atomic model diagram: Outward infection model (*OIM*).

As the population movement increase is a significant factor in the spread of the infection, it is essential to reflect this phenomenon in the simulation model [48,49]. Although the β in *IIM* indirectly contains the movement within the community, it does not include the external infection. To reflect this property, we designed the outward infection model (*OIM*), which calculated the movement of people using *calculateMovingInfection*() and sends them to the other models using the *to_TM* event at the irregular interval (i.e., $t_{\mathrm{move}}$) in the *MOVE* phase of Figure 4. Equations (40)–(47) are the DEVS expressions of the *OIM*.

(40) $$OIM=<X,\;Y,S,\;\delta_{ext},\;\delta_{int},\;\lambda ,ta>$$

(41) X=∅;

(42) Y={to_TM}

(43) S={MOVE,  STOP};

(44) $$\delta_{ext}:\emptyset ;$$

(45) $$\delta_{int}:\left(MOVE\right)\to \left(MOVE\right)\;\mathrm{if}\;t_{e}<t_{finish},\;\left(SSD\right)\to \left(RSD\right)\;\mathrm{and}\;\mathrm{execute}\;\mathrm{simulationStop}()\;\mathrm{if}\;t_{e}==t_{finish};$$

(46) λ:(MOVE)→(to_TM) and execute calculateMovingInfection()

(47) $$ta:\left(MOVE\right)\to t_{move},\;\left(STOP\right)\to \infty .$$

Based on the movement location data of subscribers provided by the mobile carrier [50,51], this study defined Equations (51)–(53) to infer the number of susceptible ($S_{ij}$), infected ($I_{ij}$), and recovered ($R_{ij}$) population moving from major city *i* to *j*. In these equations, M means the average number of movement of people to another city per day; $r_{mc}$ means the ratio of total city movement to another major city. The data of mobile carriers indicates that M and $r_{mc}$ are 0.028 and 0.076, respectively. Considering the situation in which people move from major city *i* to *j*, the number of the population entering major city *j* can be defined under the assumption that the proportion of the incoming population is the same across the entire area. Furthermore, assuming that the amount of movement is proportional to the population of the central city, the amount of population sent from major city *i* to *j* is equal to $M\times r_{mc}\times N_{j}\times N_{i}/\sum \left(N-N_{j}\right)$. Considering the sending population type is proportional to the city’s current state, as shown in Equations (48)–(50), each movement amount can be expressed as Equations (51)–(53) to note sending population type. The *IIM* calculates the $S_{ij}$, $I_{ij}$, and $R_{ij}$ via the *calculateMovingInfection()* and sends them to the *TM* with the output port *to_TM*.

(48) $$r_{s}=\frac{S_{m}\left(t\right)}{S_{m}\left(t\right)+I_{m}\left(t\right)+R_{m}\left(t\right)}\;$$

(49) $$r_{I}=\frac{I_{m}\left(t\right)}{S_{m}\left(t\right)+I_{m}\left(t\right)+R_{m}\left(t\right)}\;$$

(50) $$r_{R}=\frac{R_{m}\left(t\right)}{S_{m}\left(t\right)+I_{m}\left(t\right)+R_{m}\left(t\right)}\;$$

(51) $$S_{ij}=M\times r_{mc}\times N_{j}\times \frac{N_{i}}{\sum N-N_{j}}\times r_{s}\;$$

(52) $$I_{ij}=M\times r_{mc}\times N_{j}\times \frac{N_{i}}{\sum N-N_{j}}\times r_{I}\;$$

(53) $$R_{ij}=M\times r_{mc}\times N_{j}\times \frac{N_{i}}{\sum N-N_{j}}\times r_{R}\;$$

### 3.3. Model Identification and What-If Analysis

**Figure 5:**
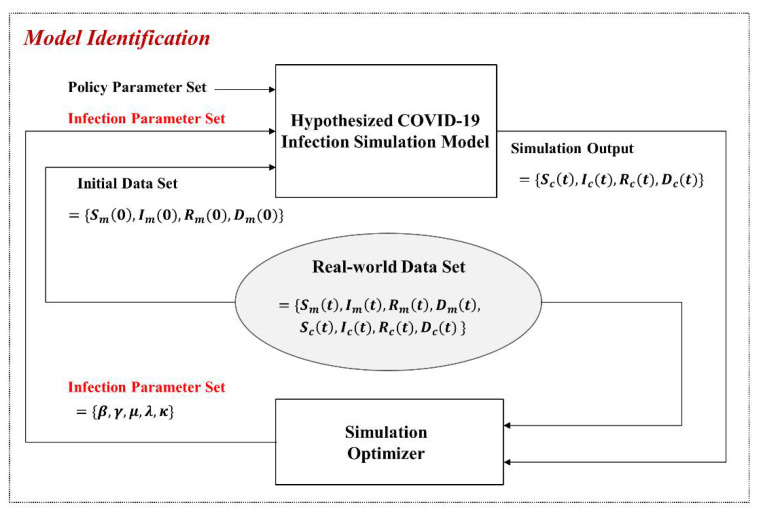
Model identification with simulation-based optimization.

Although we designed the simulation model, the model included logical behaviors but did not reflect the real-world parameters. Figure 5 shows the process to update the parameters of the model based on real-world data, which we call model identification. 

From the real world, we can acquire the S(t), I(t), R(t), and D(t) and the policy parameter set, but we cannot directly know the infection parameter set related to the *SIRD executeExtendSIRD*() and can identify it through simulation optimization, including optimization algorithms. At first, the hypothesized model is executed using the initial data set, known policy parameter set, initialized infection parameter set, and the S(t), I(t), R(t), and D(t). Next, the simulation optimizer compares the real-world data set and simulation output and updates the infection parameter set, iteratively simulating until the error converges to an acceptable range. We call the hypothesized model the updated last parameter set identified model. 

After the model identification, we can conduct a new analysis of the simulation output against the policy parameter set, which is fixed at the model identification step. The process of model identification finds the unknown part of the entire parameter set; the process of what-if analysis conducts a simulation by changing a part of the entire parameter set.

## 4. Simulation Experiment

This section confirms that the implemented simulation-based model reflects the real-world infection trend and changes in government policy or infection coefficient.

We conducted the experiments using real-world data for 160 days from 6 May 2020 to 12 October 2020. The start day was when South Korea’s distance policy began, and the final day was when the distance policy was upgraded to the second stage. 

Simulation results were optimized using the Matlab R2018 version. The algorithm used for optimization was a genetic algorithm. The desktop CPU used in the experiment was a 3.7 GHz Intel i7-8700 K processor, and the memory was 32 GB RAM. The simulator used DEVSim++ and was developed in C++.

### 4.1. Simulation-Based Model Identification

We define the infection coefficients at the model design step and predict the infected population through model simulation. Because the simulation results regarding the infected population are different from the real-world data, infection coefficients should be optimized to reflect real-world trends and applied to the simulation model. In this study, we use a genetic algorithm to find SIRD coefficients and analyze infection trends, especially social distancing effects. The optimization phase consists of three steps: (1) error calculation, (2) offspring generation, and (3) mutation generation. 

First, we define a value error by a difference between the real-world data of the COVID-19 infected population and our simulation results. We use root mean square error (RMSE) to measure the error quantitatively. The error scale is adjusted to the original scale; thus, it is easy to interpret the error results with simple numbers. Next, for the progeny generation step, the SIRD value is calculated by generating progeny using a part of the parent chromosome. Finally, mutations are used to prevent convergence to local optimization points. It is created with a 0.05% chance of mutation. Equation (54) expresses this process as an equation.

(54) $$p=arg\underset{p}{\min}\frac{1}{n}\sqrt{\sum_{t=a}^{n}{\left\{f\left(t\right)-y_{real}\left(t\right)\right\}}^{2}}$$

**Figure 6:**
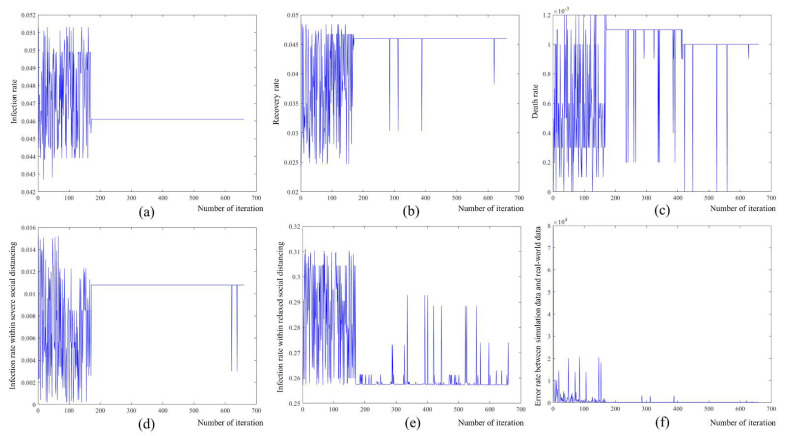
Changes of model parameters via simulation-based optimization with real-world data: (**a**) infection rate; (**b**) recovery rate; (**c**) death rate; (**d**) infection rate within severe social distancing period; (**e**) infection rate within relaxed social distancing period; (**f**) error rate between simulation data and real-world data.

Figure 6 shows the coefficients generated in the process of optimization. The x-axis represents the number of iterations performed, and the y-axis represents the value of each item. Figure 6a–e shows the SIRD coefficient and social distancing policy coefficient used in this study, and Figure 6f is the process of optimizing the above parameters and indicates the error that has occurred. Table 4 shows the optimized infection coefficients.

Genetic algorithms pass their genes to the next generation to find solutions that fit well. This process finds an optimal solution by repeating the process of generating chromosomes and calculating the fitness of the offspring. Because of this process, the value of each coefficient in Figure 6 fluctuates and then converges to an optimum value. The optimization process was conducted 660 times and then converged.

**Figure 7:**
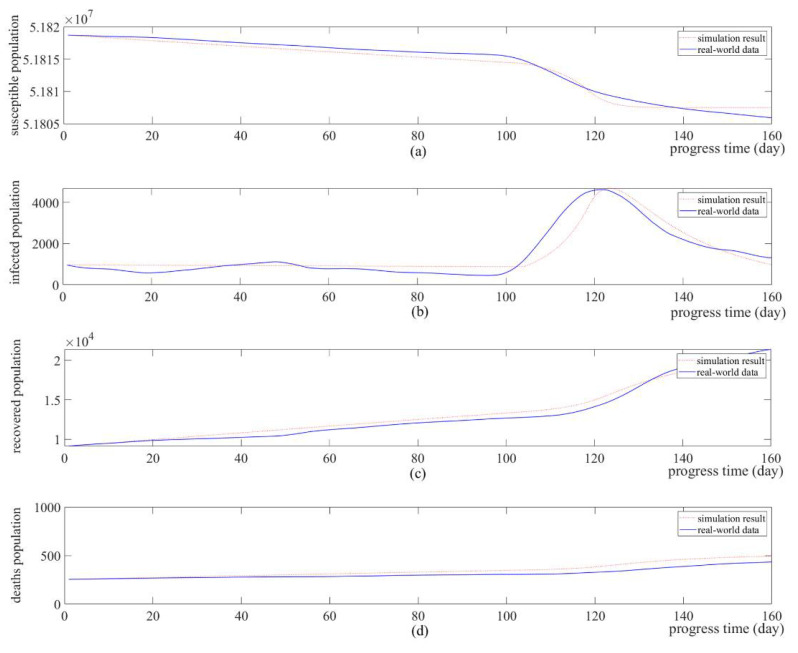
Comparison of simulation results and real-world data. (**a**) number of susceptible population; (**b**) number of infected population; (**c**) number of recovered population; (**d**) number of deaths population.

Table 4 shows the results of the optimization: the optimized infection coefficients. Figure 7 shows the results of comparing the simulation results with the SIRD values of South Korea by using the optimized infection coefficients. These simulation results show a similar trend to the real SIRD figures in South Korea, meaning that the implemented simulation-based model reflects the real-world environment well.

βγμλκFigure 7a,c,d shows no significant difference compared to the real-world data. However, Figure 7b has a more significant error than the other graphs because the infection rate beta is approximated by an exponential function according to the social distancing policy, as shown in Equations (38) and (39). Thus, real-world data with multiple slopes can be accurately calculated. Therefore, it cannot be approximated.

### 4.2. What-If Analysis: Simulation of Schedule Change in Social Distancing

**Figure 8:**
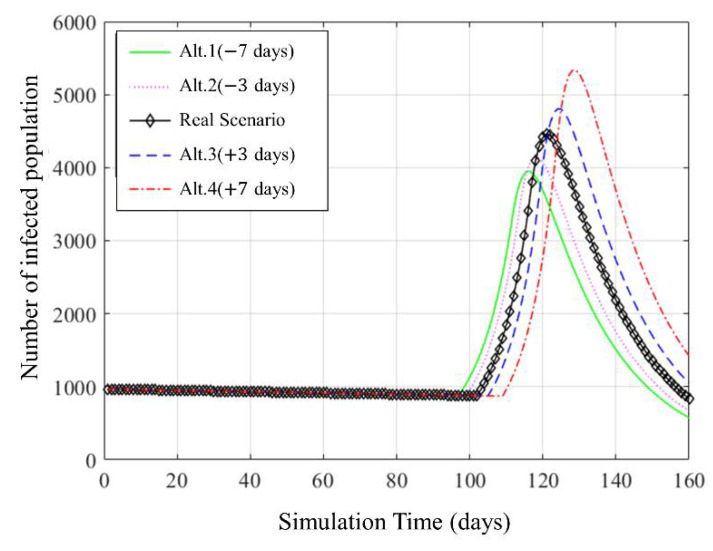
Infected population by varying start time for social distancing policy.

The what-if analysis is utilized to optimize infection coefficients in Section 4.1. It shows a comparison of each scenario in which the social distancing policy start date changes. Figure 8 shows the simulation result using the optimized infection coefficient. In this graph, the x-axis represents the simulation time, and the y-axis shows the number of infected people. Alt.1 and Alt.2 represent simulating a scenario in which the starting point of social distancing is advanced by seven days and three days, respectively, and Alt.3 and Alt.4 are when the starting point of social distancing is delayed. The maximum number of infected people and the period of maintaining social distancing are different for each scenario, depending on when social distancing is started.

**Figure 9:**
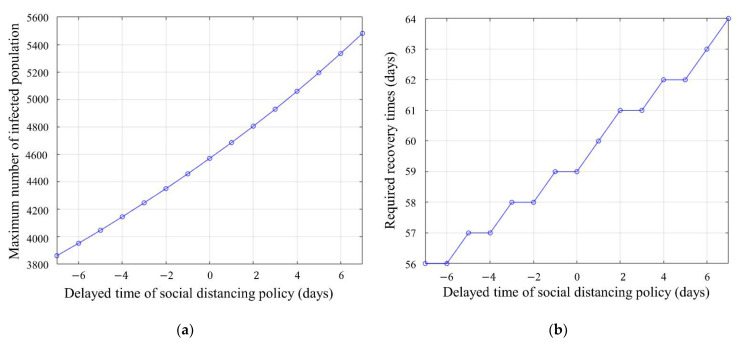
Relationship between infected population and distance policy: (**a**) maximum number of infection population by varying social distancing period; (**b**) required recovery time by varying social distancing period.

Figure 9a shows the change in the maximum number of infected people that occurs as the starting point of the social distancing policy is changed based on the results confirmed in Figure 8. In the Alt.1 scenario, where social distancing was conducted seven days earlier, up to 3800 people were infected. In Alt.4, which implemented a social distancing policy seven days later, a maximum of 5481 people were infected. Thus, if the social distancing policy is postponed for one day, an additional 250 people could be infected every day. As the simulation data are approximated by an exponential function, if the social distancing policy is delayed further, such as 15 days or 30 days, more infections can occur. On the other hand, if social distancing policies are implemented faster, more infections can be prevented. This shows that the implementation of the distancing policy is closely related to the number of infected people.

Figure 9b represents the period from which the social distancing policy is initiated until the recovery. In Figure 9b, If the social distancing policy is implemented seven days earlier, reaching the sedation period is 56 days. If the social distancing policy is implemented seven days later, it is 64 days to reach the sedation period. The sedation phase has a close relationship with the maximum number of infected populations, as the infected population must reach the threshold of the sedation phase. By delaying the implementation period of social distancing, the maximum infected population increases, and the period of social distancing policy increases. On the other hand, if social distancing is performed in advance, the maximum infected population and the social distancing period can be shortened. 

### 4.3. Discussion

We designed a hybrid model using DEVSim++ and optimized the simulation model based on Korean data. The what-if analysis experiment in Section 4.2 was conducted through the designed model. The experiment can check the trend of the COVID-19 infected population through the interaction between simulation models that include *MCM* and *TM*. *MCM* calculates SIRD using infection parameters. It also physically sends the number of moving persons between the cities to the *TM*. *TM* transmits the infected population received from the *MCM* to a pre-determined destination to spread the infection.

Here, we discuss two further applications for the developed simulators. First, The simulator overcomes the limitations of the existing standalone SIRD model by modeling the infected population between cities through the *TM*. The simulator overcomes the limitations of the existing standalone SIRD model by modeling the infected population between cities through the *TM*. In the experimental stage, optimization was performed using statistical data sets of daily and monthly infected populations in major cities in South Korea. Therefore, the infection coefficient used in this study is applied only to the case of South Korea. However, as long as there is a data set of the country you want to apply, it can be commonly applied to all countries.

Next, it shows the correlation between the maximum number of infected people and the length of time to reach recovery. The simulator of this study can infer the recovery period from the maximum infected population according to the time of distance as shown in Figure 9. As a result, it shows that it can be a reference for social distancing policy. 

## 5. Conclusions

The proposed method includes two sections: (1) hybrid modeling and (2) simulation-based optimization. The form describes the interaction among large-scale cities by integrating the existing differential equation-based model and agent-based model. The latter makes the model reflect the real world by updating the inner states of the hybrid model through the use of public data.

The proposed model simulates the number of suspected, infected, recovered, and deceased COVID-19 patients in South Korea using the SIRD infection coefficient and policy coefficient. The model is optimized with the results of the SIRD model output from the previous step to fit the data of the real-world SIRD model. In the last step, as a what-if analysis, we used the policy coefficient to simulate pulling and delaying the Korean government’s distance policy enforcement, and we experimented to confirm the result.

We expect that the proposed work plays a principal role in analyzing how social distancing effectively affects virus prevention and provides a simulation environment for the biochemical field. 

## Figures and Tables

**Table 2:** Input parameter description of simulation model.

Input Parameter	Notation	Description
Infection parameter set	β	The rate of infection
	γ	The rate of recovery
	μ	The rate of death
	λ	The attenuation coefficient of increase in the rate of infection
	κ	The attenuation coefficient of decrease in the rate of infection
Policy parameter set	$t_{rsd}$	The duration time of the relaxed social distancing policy
	$t_{ssd}$	The duration time of the severe social distancing policy

**Table 3:** Output parameter description of the simulation model.

Output Parameter	Notation	Description
Variables for SIRD model	$S_{m}\left(t\right)/S_{c}\left(t\right)$	The number of susceptible population in the major city/country at time t
	$I_{m}\left(t\right)/I_{c}\left(t\right)$	The number of the infected population in the major city/country at time t
	$R_{m}\left(t\right)/R_{c}\left(t\right)$	The number of recovered population in the major city/country at time t
	$D_{m}\left(t\right)/D_{c}\left(t\right)$	The number of deaths population in the major city/country at time t

**Table 4:** Optimized infection coefficients.

Parameter	Optimized Coefficient
β	0.0461
γ	0.0460
μ	0.0010
λ	0.0108
κ	0.2573

## Data Availability

Data available on request due to restrictions.

## Abbreviations

ABM	Agent-Based Model
D	Dead
DEVS	Discrete Event System Specifications
EBM	Equation-Based Model
EIC	External Input Coupling Relation
EOC	External Output Coupling Relation
I	Infected
IC	Internal Coupling Relation
IIM	Inner Infection Model
ISM	Infection Simulation Model
MCM	Major City Model
OIM	Outward Infection Model
R	Recovered
RMSE	Root Mean Square Error
RSD	Relaxed Social Distancing
RSD_S	Relaxed Social Distancing Steady State
RSD_T	Relaxed Social Distancing Transient State
S	Susceptible
SEIR	Susceptible, Exposed, Infected, and Recovered
SIR	Susceptible, Infected, and Recovered
SIRD	Susceptible, Infected, Recovered, and Dead
SEIR	Suspected, Exposed, Infected, and Recovered
SSD	Severe Social Distancing
SSD_S	Severe Social Distancing Steady State
SSD_T	Severe Social Distancing Transient State
TM	Transfer Model
